# Ulcer Disease in the Excluded Segments after Roux-en-Y Gastric Bypass: a Current Review of the Literature

**DOI:** 10.1007/s11695-020-05123-w

**Published:** 2020-11-24

**Authors:** Gabriel Plitzko, Grégoire Schmutz, Dino Kröll, Philipp C. Nett, Yves Borbély

**Affiliations:** Clinic for Visceral Surgery and Medicine, University Hospital Bern Inselspital, and University of Bern, Freiburgstrasse, CH-3010 Bern, Switzerland

**Keywords:** Anti-inflammatory agents, Non-steroidal anti-inflammatory drugs, Roux-Y gastric bypass, Peptic ulcer perforation, Hemorrhage, Gastritis, Gastrostomy, Morbid obesity, Gastric remnant

## Abstract

Ulcer disease in excluded segments after Roux-Y gastric bypass (RYGB) is rare but can evolve into a life-threatening situation. The excluded segments exhibit a different behavior from that of non-altered anatomy; perforated ulcers do not result in pneumoperitoneum or free fluid, and therefore must be met with a low threshold for surgical exploration. The anatomical changes after RYGB impede routine access to the remnant stomach and duodenum. There are various options to address bleeding or perforated ulcers. While oversewing and drainage preserves the anatomy and forgoes resection, remnant gastrectomy offers a definitive solution. The importance of traditional risk factors such as smoking or use of non-steroidal anti-inflammatory drugs is unclear. Eradication of *Helicobacter pylori* and secondary prophylaxis with proton-pump inhibitors is advisable, albeit in double-dose.

## Introduction

Bariatric surgery is the most effective treatment for morbid obesity and results in sustained weight loss and resolution of comorbidities. Over the past decades, the number of performed bariatric operations has been on a constant rise, reaching more than half a million procedures a year worldwide [1]. This number is expected to increase even further, as metabolic surgery is advocated as a treatment option for type 2 diabetes mellitus (T2DM) in patients with a body mass index (BMI) < 35 kg/m^2^ [2].

Roux-en-Y gastric bypass (RYGB) is one of the most commonly performed procedures, is seen by many as the gold standard treatment in patients with preexisting gastroesophageal reflux disease, and is suggested as the first-choice procedure for patients with T2DM [1]. After the creation of a small gastric pouch, food is diverted away from the stomach directly into the small bowel, and acid produced in the remnant stomach will no longer reach the esophagus anymore. These distinctive changes in anatomy are thought to be key factors for sustained weight loss. However, they also result in the downsides of this procedure, above all hindering access to the biliary tree, the remnant stomach, and the duodenum [3–5].

Apparent peptic ulcer disease (PUD) in the excluded stomach or duodenum is rare but leads to severe morbidity and can be life-threatening. The anatomical changes post-RYGB lead to a different clinical presentation in an altered pathophysiological environment [6]. Whereas there are extensive data on marginal ulceration (MU)—ulcers at the gastro-jejunal anastomosis—experience with ulceration in the excluded segments, especially in regard to treatment and prevention, is mostly limited to small series and case reports [6–28]. Therefore, this review aims to summarize the available data, to contrast existing guidelines to the altered situation post-RYGB and provide treatment recommendations.

## Materials and Methods

**Fig. 1:**
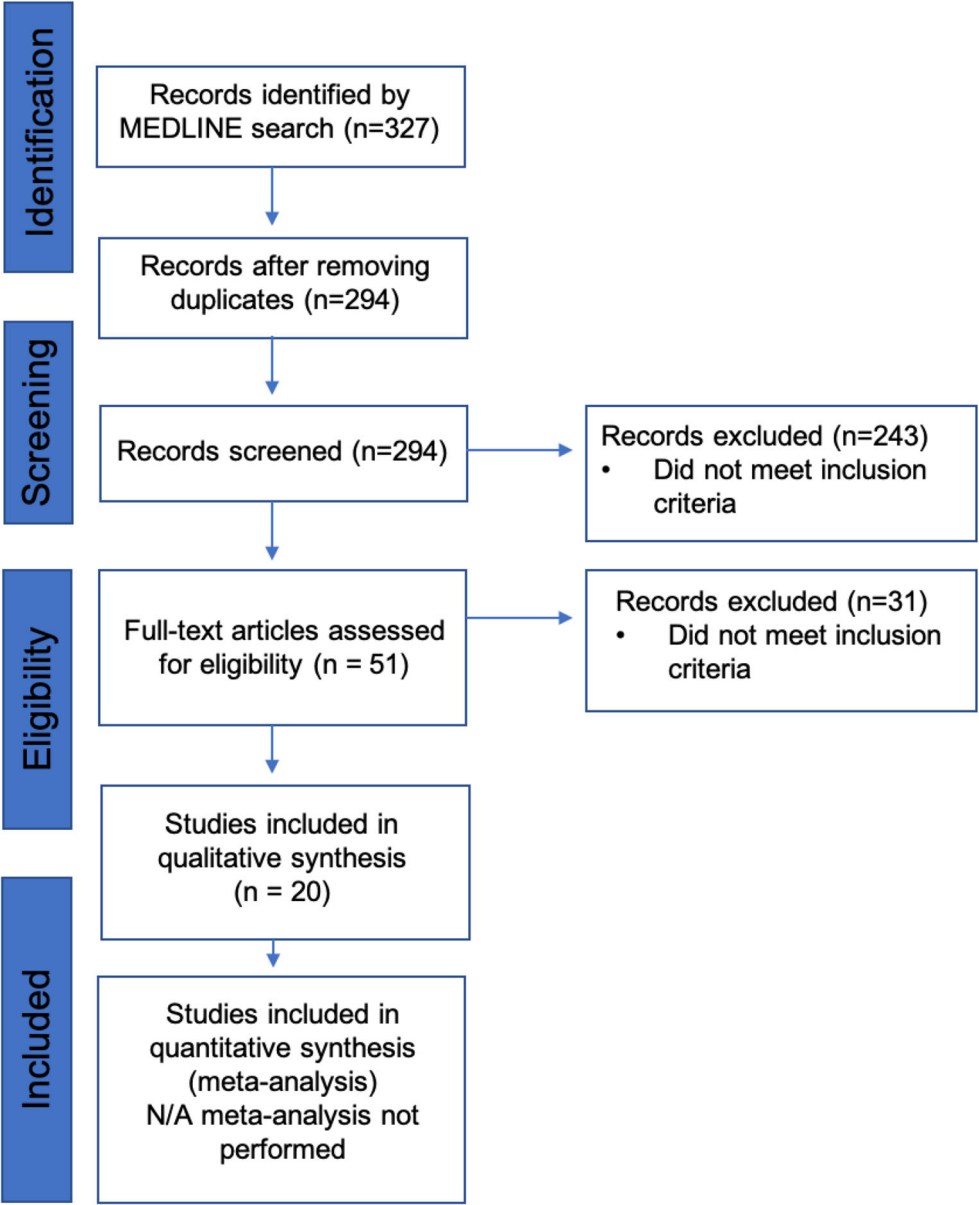
Flow diagram of study selection [29]

All studies reporting ulcer formation in the excluded segments of post-RYGB patients were considered eligible. A MEDLINE search was performed during May 2020. The search terms applied are listed in Table 1. In addition, the reference list of articles retrieved by the search was assessed for further publications. Titles and abstracts of publications were screened by the primary author GP. Full texts of possibly relevant studies were evaluated to determine eligibility. All eligible articles were reviewed by the corresponding author YB and co-author PN. Consensus among authors was made regarding decisions to exclude articles. Non-English articles were also excluded. The PRISMA flow diagram is presented in Fig. 1 [29].

**Table 1:** Search terms and retrieved results

Search set	Terms	Results
#1	“gastric bypass”[Title/Abstract] AND ulcer[Title/Abstract] NOT marginal[Title/Abstract]	151
#2	“gastric bypass”[Title/Abstract] AND perforation[Title/Abstract] NOT anastomosis[Title/Abstract]	115
#3	“gastric bypass”[Title/Abstract]) AND “gastrointestinal bleeding”[Title/Abstract] NOT anastomosis[Title/Abstract]	47
#4	“gastric bypass”[Title/Abstract]) AND “gastrointestinal hemorrhage”[Title/Abstract] NOT anastomosis[Title/Abstract]	14

**Table 2:** Patient characteristics and risk factors (*Y* yes, *N* no, *n/a* not available, *NSAIDs* non-steroidal anti-inflammatory drugs)

Author [Ref]	Patient characteristics				Risk factors	
	Sex (F/M)	Age (years)	Time since operation (years)	Smoking (Y/N)	NSAIDs (Y/N)	*H. pylori*
Andersen [7]	1/0	34	3	n/a	N	n/a
Arshava [11]	0/2	36	3	n/a	Y	n/a
		54	0.2	n/a	n/a	n/a
Bjorkman [12]	0/1	24	6	n/a	N	n/a
Braley [13]	2/1	49	17	n/a	n/a	n/a
		59	17	n/a	n/a	n/a
		49	16	n/a	n/a	n/a
Dai [9]	1/0	54	5	N	N	neg
Eid [14]	0/1	61	10	N	N	n/a
Gypen [15]	1/0	35	0.2	n/a	n/a	pos
Husain [16]	1/0	63	1.5	n/a	n/a	n/a
Iranmannesh [28]	4/3	48	20	n/a	Y	empirical eradication
		39	10	n/a	N	empirical eradication
		61	7	n/a	N	empirical eradication
		54	10	n/a	N	empirical eradication
		40	13	n/a	N	empirical eradication
		70	9	n/a	N	empirical eradication
		45	16	n/a	Y	empirical eradication
Iskandar [17]	0/2	59	10	n/a	N	neg
		37	n/a	n/a	N	neg
Issa [18]	0/1	39	1.2	n/a	N	pos
Ivanecz [19]	0/1	59	2	N	Y	neg
Macgregor [20]	9/2	63	1.9	n/a	N	n/a
		37	1.8	n/a	N	n/a
		40	8	n/a	N	n/a
		31	0.6	n/a	N	n/a
		53	5	n/a	N	n/a
		43	8	n/a	N	n/a
		29	11	n/a	N	n/a
		48	4	n/a	N	n/a
		57	1.5	n/a	N	n/a
		40	20 days	n/a	N	n/a
		56	12	n/a	N	n/a
Mittermair [21]	1/0	54	1.3	n/a	n/a	pos
Ovaere [10]	1/0	33	1.2	N	N	neg
Papasavas [22]	1/0	35	1	n/a	n/a	neg
Patrascu [8]	1/0	52	7	N	N	neg
Pohl [6]	2/0	46	6	N	N	neg
		74	14	N	N	neg
Sasse [26]	1/1	55	1	n/a	Y	n/a
		47	3.5	n/a	Y	n/a
Snyder [23]	4/2	53–67	1.5–6	n/a	N	1 pos; 2 neg; 3 n/a
Spires [24]	0/1	48	4	n/a	n/a	n/a
Zagzag [27]	5/0	57	5	n/a	n/a	pos
		54	12	n/a	n/a	pos
		53	9	n/a	n/a	neg
		47	0.7	n/a	n/a	n/a
		35	2	n/a	n/a	neg
Zerey [25]	0/1	57	12	N	N	n/a

**Table 3:** Presentation, treatment, and outcomes

Author	*n*	Presentation	Location	Treatment	Outcome	Follow-up
Andersen [7]	1	Perforation	Gastric remnant	Ulcer excision, bypass take down	Cured	9 days
Arshava [11]	2	Perforation	Gastric remnant	Remnant gastrectomy	Cured	6 months
		Perforation	Gastric remnant	Remnant gastrectomy, pancreas preserving duodenal resection	Cured	n/a
Bjorkman [12]	1	Perforation	Duodenum	Remnant gastrectomy, duodenal stump oversewing	Cured	n/a
Braley [13]	3	Bleeding	Duodenum	Remnant gastrectomy, resection of first portion of duodenum	Cured	24 months
		Bleeding	Gastric remnant	Remnant gastrectomy	Cured	12 months
		Bleeding	Gastric remnant	Remnant gastrectomy	Cured	9 months
Dai [9]	1	Perforation	Gastric remnant	Oversewing, omental patch, gastric tube placement	Cured	4 weeks
Eid [14]	1	Bleeding	n/a	Angiography, coiling	Cured	n/a
Gypen [15]	1	Perforation	Duodenum	Oversewing, omental patch	Cured	6 months
Husain [16]	1	Bleeding	Duodenum	Remnant gastrectomy, resection of first portion of duodenum	Cured	n/a
Irannmanesh [28]	7	Perforation	Gastric remnant	Oversewing, omental patch	Cured	12 days
		Perforation	Gastric remnant	Oversewing, omental patch	Cured	3 days
		Perforation	Gastric remnant	Oversewing, omental patch, gastrostomy	Cured	8 dyas
		Necrosis	Gastric remnant	Remnant gastrectomy	Cured	8 days
		Bleeding	Gastric remnant	Enteroscopy with local hemostasis	Cured	5 days
		Bleeding	Gastric remnant	conservative	Cured	3 days
		Bleeding	Gastric remnant	Enteroscopy with local hemostasis	Cured	3 days
Iskandar [17]	2	Perforation	Duodenum	Oversewing	Cured	1 week
		Perforation	Duodenum	Duodenostomy, drainage	Cured	n/a
Issa [18]	1	Bleeding	Duodenum	Laparoscopic gastroduodenoscopy, electrocoagulation	Cured	10 months
Ivanecz [19]	1	Bleeding	Duodenum	Remnant gastrectomy, resection of first portion of duodenum	Cured	3 months
Macgregor [20]	11	Perforation	Duodenum	Oversewing, subsequent remnant gastrectomy	Cured	n/a
		Perforation	Gastric remnant	Oversewing and gastrotomy, subsequent remnant gastrectomy	Cured	n/a
		Perforation	Duodenum	Oversewing	Cured	n/a
		Perforation	Duodenum	Oversewing, and gastrotomy, subsequent remnant gastrectomy	Cured	n/a
		Perforation	Duodenum	Oversewing, vagotomy, pyloroplasty	Cured	n/a
		Perforation	Duodenum	Oversewing, subsequent remnant gastrectomy	Cured	n/a
		Perforation	Duodenum	Oversewing, subsequent remnant gastrectomy	Cured	n/a
		Perforation	Duodenum	Oversewing, subsequent remnant gastrectomy	Cured	n/a
		Perforation	Gastric remnant,	Oversewing, subsequent remnant gastrectomy	Cured	n/a
		Perforation	Duodenum	Oversewing, subsequent remnant gastrectomy	Cured	n/a
		Perforation	Duodenum	Gastrostomy, subsequent remnant gastrectomy	Cured	n/a
Mittermair [21]	1	Perforation	Duodenum	Oversewing	Cured	6 days
Ovaere [10]		Perforation	Gastric remnant	Oversewing, omental patch	Cured	n/a
Papasavas [22]	1	Perforation	Gastric remnant	Remnant gastrectomy	Cured	38 months
Patrascu [8]		Bleeding	Gastric remnant	Remnant gastrectomy	Cured	2 months
Pohl [6]	2	Perforation	Duodenum	Oversewing, omental patch	Cured	3 years
		Perforation	Duodenum	Oversewing, omental patch	Cured	3 years
Sasse [26]	2	Perforation	Gastric remnant	Oversewing, omental patch	Cured	2 years
		Perforation	Gastric remnant	Intraoperative death	Death	
Snyder [23]	6	4 perforation	4 duodenum	5 remnant gastrectomy	5 Cured	2–4 years
		2 Bleeding	2 Gastric remnant	1 duodenal oversewing	1 Death	
Spires [24]	1	Bleeding	Duodenum	Remnant gastrectomy	Cured	6 months
Zagzag [27]	5	Perforation	Duodenum	Graham patch	Cured	n/a
		Perforation	Duodenum	Graham patch	Cured	n/a
		Perforation	Duodenum	Graham patch	Cured	n/a
		Perforation	Duodenum	Graham patch	Cured	n/a
		Perforation	Duodenum	Graham patch	Cured	n/a
Zerey [25]	1	Bleeding	Duodenum	Duodenotomy, oversewing	Cured	5 days

The following data were collected: age, sex, time since the bariatric procedure, potential risk factors (smoking behavior, NSAIDs, HP), clinical presentation, radiological findings, site of affection, treatment, outcome, and duration of follow-up.

## Incidence and Presentation

The true incidence of PUD after RYGB is not known, and the reported cases are certainly biased towards complicated PUD, such as bleeding and perforation.

Here, we summarize reports of 54 patients in 5 case series and 18 case reports (tbl1, tbl2) [6–28]. Thirty-five (65%) of these individuals were female with an age ranging from 21 to 74 years. The interval between surgery and the onset of symptoms varied between 2.5 months and 20 years (Table 2). Fifteen patients (28%) presented with gastrointestinal bleeding, whereas 38 patients (70%) had perforated ulcers. The site of bleeding was the gastric remnant in 53% and the duodenum in 47%; 34% of perforations were localized in the gastric remnant and 66% were in the duodenum (Table 3). The leading symptom at presentation was epigastric or upper abdominal pain. In cases of bleeding, melena, weakness, and anemia were common findings. Patients with perforation presented with signs of sepsis, such as hypotonia, tachycardia, fever, and elevated leukocytes.

## Pathophysiological Considerations

### Gastrin Levels and Acid Production in the Excluded Stomach

After RYGB, there is continued, although diminished, basal, and stimulated acid excretion in the excluded stomach. Gastrin levels are decreased after RYGB, yet the gastric mucosa maintains its ability to respond to vagal and hormonal stimuli [30], thus preserving an acidic environment [12, 30–32]. The amount of acid production is influenced by the proportion of the parietal cell mass, partitioned by surgery to the pouch and the distal stomach, a factor certainly influencing the development of MU [33–35]. In theory, high transection increases acid production in the excluded stomach and contributes to acid-related mucosal injury in the antrum and duodenum [12, 31]. Furthermore, post-RYGB, cellular hypertrophy of the gastric mucosa occurs in the presence of a reduction in G cells, again lowering gastrin production [36]. Additionally, in proton-pump inhibitor (PPI)–treated mice with normal anatomy, the resulting hypochlorhydria causes bacterial overgrowth resulting in gastric inflammation [37].

### Buffering Effect of Nutrients

After RYGB, there is no counteracting effect on the acid in the antrum and duodenum as the ingested food bypasses the excluded segments. However, the exclusion of the duodenum also leads to a reduced and desynchronized pancreatic secretion as the strongest stimulus of bicarbonate secretion is the duodenal presentation of nutrients. Furthermore, bile refluxing into the gastric remnant also contributes to mucosal injury [12, 21, 38, 31].

### Role of PPI

In large database-driven studies, up to a quarter of post-RYGB patients are on PPIs [39, 40]. There is an interplay between PPI intake and gastrin levels, and long-term use leads to hypergastrinemia stimulated by a reduction in gastric acid secretion via somatostatin feedback [41]. RYGB results in altered gastric emptying, reduction in intestinal absorption surface, and a change in pH. This leads to pharmacokinetic changes where PPIs, as lipophilic drugs, are absorbed to a lesser degree [42]. As PPIs are degraded by luminal acid, they are usually protected by a coating to prohibit premature activation and enable later absorption in the small bowel which again is altered. Likewise, opened PPI capsules have a significantly better effect on MU healing than intact capsules [43]. In line with these pharmacokinetic changes, plasma concentrations of omeprazole metabolites in patients after RYGB are significantly reduced compared to the corresponding levels in pre-RYGB and controls [30].

### Other Risk Factors: NSAIDs, Smoking, *H. pylori*, and ZES

#### Nonsteroidal Anti-inflammatory Drugs

More than 30 million patients use NSAIDs on a daily basis [44]. As unselective inhibitors of the cyclo-oxygenase pathway, they deeply affect the gastric mucous barrier [44]. NSAID use is a risk factor for PUD in the general population and for MU after RYGB [45, 46]. In the long term, up to 40% of NSAID users experience gastroduodenal ulcers [12].

In 40 of the 54 reported patients (74%), NSAID use was stated. Surprisingly, only 6 of the 40 patients (15%) had a history of NSAID use. Thus, other factors might have a higher impact on ulcer formation.

#### Smoking

There is wide evidence implicating smoking as a risk factor for the occurrence, recurrence, and complications of PUD [47]. Suggested mechanisms include increased gastric acid secretion, altered gastric motility, a higher rate of duodenogastric reflux, and impaired duodenal and pancreatic bicarbonate secretion [47, 48]. Among patients with RYGB, smoking seems to increase the risk for MU and especially perforation [49]. Furthermore, ulcer healing is impaired in smoking patients compared to non-smokers [49].

Smoking behavior was reported only for 8 patients (15%), and none of them currently smoked.

#### *Helicobacter pylori*

HP (*Helicobacter pylori*) is one of the most common human infections and plays a pivotal role in the development of gastritis, ulcer formation, and malignant lesions [50–53]. Its prevalence in bariatric patients ranges up to 85%, yet data are controversial and cover mostly MU [54]. Consequently, international guidelines differ in their recommendations for HP screening and management in bariatric patients [55, 56].

Detection of HP in post-RYGB patients can be challenging; histological samples remain the gold standard. Urea breath tests, especially in the absence of a pouch infection, can be falsely negative, as most of the gastric mucosa will not be in direct contact with urea [15]. Serology is of less diagnostic value since complete resolution of elevated IgG antibody titers after treatment of HP infection is not common, even though negative IgG antibodies can exclude HP [50, 57]. With a sensitivity and specificity over 90%, monoclonal stool antigen tests are probably the most suitable non-invasive diagnostic tool [50, 57].

Reported data provided the HP status for 20 of 54 patients (37%). Based on histological findings, 7 (15%) were positive. However, 7 more were eradicated empirically. Given the unknown influence of HP on the development of PUD after RYGB, its role as the most common proven risk factor for gastric cancer, and impaired access to the excluded stomach and duodenum for surveillance, patients should be screened preoperatively unless further data are available [55, 56]. Additionally, biopsies should be taken in revisional cases.

#### Zollinger-Ellison Syndrome (ZES)

ZES (Zollinger-Ellison syndrome) is a rare condition in which gastrin-secreting neuroendocrine tumors cause elevated acid production from gastric parietal cells, thus leading to recurrent peptic ulcers. Approximately 75% of ZES are sporadic, whereas 25% are associated with multiple endocrine neoplasm type 1. There is a single case report of a patient with recurrent gastrojejunal anastomotic strictures and MU following RYGB due to a gastrinoma of the duodenum [58]. The role of ZES is negligible but should be considered in cases of multiple recurrent or refractory ulcers.

### Gastrointestinal Motility: Gastroparesis/Stasis

Gastroparesis can occur following planned or inadvertent vagotomy [59]. Its incidence after partial gastrectomy is up to 5% [60]. Changes in ghrelin secretion influence gastric motility [60, 61]. Bile reflux into the gastric remnant is observed in 36% of post-RYGB patients, and its deleterious effects on the mucosal barrier lead to chronic gastritis and intestinal metaplasia and may contribute to the formation of ulcers [62, 63].

Furthermore, delayed emptying and stasis in the biliopancreatic limb may predispose patients to bacterial overgrowth followed by inflammatory changes in the intestinal wall potentially leading to ulceration [64].

## Diagnosis

### Incidence of Gastritis and Predictors of PUD

Studies evaluating the gastric remnant after RYGB are rare. Among 53 patients with RYGB, remnant gastritis was found in 87% of patients with a normal mucosa in the pouch, indicating a harmful effect of unbuffered acid on the gastric remnant [31]. Elsewhere, in more than half of patients taking PPI, endoscopy of the gastric remnant revealed peptic changes [30].

Considering symptoms as surrogates for gastritis and PUD, upper abdominal pain by far is the most frequent symptom leading to readmissions after RYGB [65]. Of the reported patients, over two-thirds reported epigastric and/or upper abdominal pain.

### Free Air: Plain X-ray and Abdominal CT

With a sensitivity of approximately 86% in detecting gastrointestinal perforation, CT is widely accepted as accurate in the evaluation of such patients [66]. However, in post-RYGB patients, the presence of free intra-abdominal air due to perforation of the excluded segments is much less common if not absent. Patients with perforation had plain X-rays in 20 of 41 cases (49%), and free air was detected in two patients. When performed, 44% of CT scans showed free intra-abdominal air. Thus, in patients after RYGB, negative CT findings do not exclude a viscus perforation, and operative exploration should be performed with a low threshold. Indeed, the presence of free air and even more fluid should raise suspicions about a pouch-gastric fistula or irregularities at the level of the jejuno-jejunal anastomosis [6, 15, 20, 67].

### Bleeding: CT and Mesenteric Angiography

The sensitivity and specificity of CT angiography for active gastrointestinal bleeding are over 90%. However, its sensitivity for obscure bleeding is only approximately 45% [68]. Even though it lacks therapeutic options, CT angiography provides essential information regarding the bleeding origin and allows for subsequent mesenteric angiography to be targeted to an area of interest leading to an improved angiographic detection rate. The technical success rate of transarterial embolization in gastrointestinal bleeding is reported to be up to 93% with a rate of bleeding cessation up to 81% [69].

Among the reported cases of ulcer bleeding following RYGB, only indirect signs but no active bleeding were detected. Mesenteric angiography without previous CT angiography was performed in 25% of patients, and angiography showed active bleeding in 67% of patients. One patient had successful embolization, whereas embolization failed in the other patient, and surgical intervention was needed to control bleeding [14, 25].

Transarterial angiography with subsequent embolization is an option to address bleeding in those patients. This procedure is non-invasive and has a high success rate [69]. However, in a general context, it might be more purposeful to address such patients surgically, or rather endoscopically via temporary gastrostomy.

### Access to the Excluded Segments: Double-Balloon Endoscopy, Intraoperative Temporary Gastrostomy, and Pouch Gastrostomy

There are various ways to access the excluded segments post-RYGB. Upper endoscopy is considered a first-line approach for the diagnosis and treatment of upper gastrointestinal bleeding and provides a valid alternative in the treatment of postbariatric complications such as leaks or anastomotic bleeding [70]. Whereas gastro-jejunal anastomoses can easily be evaluated, the combined length of alimentary and biliopancreatic limbs complicates access to the gastric remnants and duodenums. With the use of a pediatric colonoscope, the remnant can be reached in up to 68% of cases [31]. This rate can be raised to 88% by applying a double-balloon technique albeit with a higher perforation rate of 10% [71].

Eleven (20%) of the reported patients underwent a standard upper endoscopy, which was non-diagnostic in every case. In 5 patients, retrograde endoscopy was attempted; the remnant was reached in three patients, and treatment of the bleeding was successful in two patients [12, 14, 22, 28]. Thus, in time-critical situations, conventional upper endoscopy can rule out pathologies of the gastric pouch, anastomosis, and alimentary limb, but its diagnostic value in assessing the excluded segments is limited.

Access to the excluded segments can further be achieved with percutaneous transgastric endoscopy. CT or ultrasonographic guidance to introduce a guidewire is followed by placement of a gastrostomy tube into the remnant stomach. Serial dilations of the gastrostomy over several weeks are then required until an endoscope can be introduced. This technique is safe and effective but obviously not suitable for emergency situations [4, 5, 62].

Intraoperative transgastric endoscopy allows for addressing or excluding other pathologies at the same time [72]. Usually, access to the gastric remnant is established during laparoscopy via temporary remnant gastrostomy [72]. Among the reviewed patients with ulcer bleeding or perforation after RYGB, 8 of 54 had (15%) intraoperative transgastric endoscopy [13, 18, 25, 28]. Endoscopy revealed duodenal ulcers in 3 cases and gastritis and benign ulcers in 2 cases.

## Treatment and Follow-Up

There are various options to address complicated PUDs. The main goal is to resolve septic foci and provide a solution for future follow-up in this special anatomic situation at the same time.

### Gastrectomy Versus Oversewing Ulcers

As patients with PUD after RYGB are more likely to be diagnosed with complicated PUD, the treatment is generally operative. There are several controversial issues, such as whether to perform remnant gastrectomy, ulcer repair with concomitant vagotomy, or mandatory intraoperative gastroscopy. In the case of free air or fluid, assessing the patency of the jejuno-jejunostomy is mandatory [67].

The recurrence rate of PUD determines the extent of surgery needed. So far, the paucity of data and the rather short follow-up overall do not allow for general recommendations. Although remnant gastrectomy seems drastic, it certainly facilitates the follow-up and most effectively counteracts PUD unless there is ectopic gastric tissue elsewhere. In the case of perforated PUD, oversewing after biopsies is an option. It is certainly advisable to enable easier future access to the remnant stomach, especially when intraoperative gastroscopy is not performed at the time of the procedure. (Super)selective vagotomy may be added to reduce acid production.

Of 54 described cases, 50 (93%) underwent surgery. In 48% of these patients, remnant gastrectomy was performed, combined with resection of the first portion of the duodenum in 6% and pancreas-preserving duodenal resection in 2%. Oversewing or Graham patches were performed in 37% of patients. Other procedures included ulcer excision with bypass reversal, duodenostomy with drainage, coagulation by intraoperative endoscopy, and angiographic coiling (tbl 2).

In a short-term follow-up, 94% of patients were cured, one needed reoperation because of early postoperative bleeding following remnant gastrectomy with resection of the first part of the duodenum, and two patients died, one from pulmonary complications and the other one intraoperatively due to multiorgan failure.

There is a wide variety in the duration of follow-up, ranging from 5 days to 4 years and only limited data are available overall.

### Follow-Up: Secondary Prophylaxis, Cancer Risk

Postoperative eradication of HP was routine for positive patients. Secondary prophylaxis with PPIs was initiated in 23 of 54 patients (of whom one had a remnant gastrectomy), with varying durations, from 2 weeks to lifelong. Meta-analytic data suggest a significant benefit of prophylactic PPI in reducing MU after RYGB [73]. Despite lacking evidence, PPI may therefore be considered to counteract recurrent PUD in the excluded segments.

Cancer in the remnant stomach is rare; a meta-analysis found 17 patients who reported with pain, abdominal distension, and weight loss as main symptoms [74].

## Conclusion

PUD in the excluded segments post-RYGB is rare but life-threatening. The presentation of perforated PUD is different from that in non-bariatric patients, as the usual signs, such as free air, may not be present. Access to the excluded segments is impaired; in time-critical situations, temporary gastrostomy during laparoscopy is a viable option. Remnant gastrectomy is a definitive solution that forgoes the need for further endoscopic follow-up. Scarce available data do not allow for any conclusion about the role of potential risk factors; however, HP should be eradicated. Secondary prophylaxis with PPI should be administered in increased dosage due to altered absorption post-RYGB.
